# Automatic semantic segmentation of kidney cysts in MR images of patients affected by autosomal-dominant polycystic kidney disease

**DOI:** 10.1007/s00261-020-02748-4

**Published:** 2020-09-17

**Authors:** Timothy L. Kline, Marie E. Edwards, Jeffrey Fetzer, Adriana V. Gregory, Deema Anaam, Andrew J. Metzger, Bradley J. Erickson

**Affiliations:** 1 Department of Radiology, Mayo Clinic, Rochester, MN 55905 USA; 2 Division of Nephrology and Hypertension, Mayo Clinic, Rochester, MN 55905 USA

**Keywords:** Autosomal-dominant polycystic kidney disease, Semantic cyst segmentation, Deep learning, Magnetic resonance imaging

## Abstract

**Purpose:**

For patients affected by autosomal-dominant polycystic kidney disease (ADPKD), successful differentiation of cysts is useful for automatic classification of patient phenotypes, clinical decision-making, and disease progression. The objective was to develop and evaluate a fully automated semantic segmentation method to differentiate and analyze renal cysts in patients with ADPKD.

**Methods:**

An automated deep learning approach using a convolutional neural network was trained, validated, and tested on a set of 60 MR T2-weighted images. A three-fold cross-validation approach was used to train three models on distinct training and validation sets (*n* = 40). An ensemble model was then built and tested on the hold out cases (*n* = 20), with each of the cases compared to manual segmentations performed by two readers. Segmentation agreement between readers and the automated method was assessed.

**Results:**

The automated approach was found to perform at the level of interobserver variability. The automated approach had a Dice coefficient (mean ± standard deviation) of 0.86 ± 0.10 vs Reader-1 and 0.84 ± 0.11 vs. Reader-2. Interobserver Dice was 0.86 ± 0.08. In terms of total cyst volume (TCV), the automated approach had a percent difference of 3.9 ± 19.1% vs Reader-1 and 8.0 ± 24.1% vs Reader-2, whereas interobserver variability was − 2.0 ± 16.4%.

**Conclusion:**

This study developed and validated a fully automated approach for performing semantic segmentation of kidney cysts in MR images of patients affected by ADPKD. This approach will be useful for exploring additional imaging biomarkers of ADPKD and automatically classifying phenotypes.

## Introduction

Autosomal-dominant polycystic kidney disease (ADPKD) is the most common hereditary renal disease, affecting roughly 12 million people worldwide, and is currently the fourth leading cause of kidney failure [1, 2]. Its pathology is such that the continuous growth of cysts causes a progressive increase in total kidney volume (TKV). A typical ADPKD patient exhibits progressive renal function decline and roughly 70% progress to end-stage renal disease between age 40 and age 70 [3, 4].

TKV has been shown in a number of studies to be a useful predictor of ADPKD progression [5–7]. Similarly, the ability to delineate and measure cystic burden further contributes to our knowledge of disease progression, structure, and genotypic variances. It is well understood that the development and growth of cysts is strongly correlated with renal function decline [6, 8]. In addition, it has been shown that there is a direct correlation between TKV growth and cyst growth; however, the rate at which the cysts grow and new cysts form is dependent on each individual [9]. Furthermore, longitudinal studies have found that over time, patients with ADPKD experience an increase in TKV and cyst volume and a decrease in total parenchyma volume suggesting that the non-cystic kidney tissue is being replaced by more cysts and continuously enlarging cysts [10]. Interestingly, cyst growth and cystic index (ratio of cyst volume to TKV) varies significantly between the PKD1 and PKD2 genotypes, as patients within the PKD1 population tend to develop cysts earlier [11, 12]. Additional analysis of cystic burden and growth has the potential to inform on disease trends and therapeutic strategies.

As new imaging biomarkers emerge, scientists seek fast and efficient methods for isolating the cystic and non-cystic kidney regions for more in-depth, quantitative analysis of tissue properties [13, 14]. In the past, cyst and kidney regions have been segmented manually, which is highly labor intensive and subjective [15]. Various semi-automated cyst segmentation approaches have been proposed using intensity-based thresholding as an initialization [16, 17] as well as classical machine learning techniques such as *k*-means clustering [18], contour methods [19], and shape prior probability maps [20]. However, a fully automated deep learning approach using neural networks has the potential to rid the image analyst from the tedium of manual tracing and provide reproducible and robust volume calculations and segmentations. Deep learning is unique to the above mentioned segmentation methods in that the model is capable of “learning” important image features from the data inputs that allow it to perform its ultimate segmentation task. Through training, the model is capable of detecting patterns, pixel intensities, and shape information that may not be easily detectable to the human eye.

Convolutional neural networks (CNNs) that begin with reducing spatial resolution followed by restoration of resolution excel at pixel/voxel-level medical image segmentation tasks due to their unique architecture. In short, the first contraction section is a series of convolutional and resolution reducing layers which are used to decrease the complexity of the image and the second expansion section is essentially a mirror image of the first path used to combine feature and spatial information. The U-Net architecture [21] is one such network that has been significantly leveraged in medical image analysis to solve segmentation tasks. A particular benefit of this architecture is that it doesn’t require a large training set compared to other networks and yields highly accurate segmentation outputs.

In this study, we utilize a dataset of MR images of PKD kidneys with cyst tracings by two readers serving as ground truth. An automated approach is developed (a modified U-Net type architecture), and an ensemble model is established and tested on a test dataset. The deep neural network model described in this study allows for semantic segmentation of kidney cysts for total cyst volume (TCV) determination and may prove useful for further evaluation of disease phenotypes.

## Materials and methods

### MR image data

This retrospective study received approval from the institutional review board at https://github.com/TLKline/AutoKidneyCyst. MR scans of 60 unique patients with ADPKD of varying levels of severity were drawn from our PKD image database. T2-weighted fat (*N* = 42) and non-fat saturated (*N* = 18) scans were used in this analysis. The MR images were coronal single shot fast spin echo (SSFSE) T2 sequences, acquired with a GE scanner, with matrix size 256 × 256xZ (with Z large enough to cover the full extent of the kidneys within the imaged volume). Image voxel sizes were on the order of 1.5 mm in-plane with typically 3.0 mm slice thicknesses.

### Manual segmentations

The kidney and cyst tracings were manually performed by two image analysts (https://github.com/TLKline/AutoKidneyCyst) with years of experience performing these tracings. The training/validation set were traced by one reader, and the test set was traced by both in order to assess interobserver variability. The image analysis protocol excludes the renal pelvis and vascular structures. From the tracings, TKV and TCV were calculated as the number of voxels multiplied by the voxel volume. Each analyst was blinded to the other’s tracings. These tracings were exported as NIfTI files.

### Data stratification

From the TKV segmentations that were generated for each scan, the scans were sorted into 40 training/validation cases and 20 cases for the hold out test set. The training/validation dataset had 28 fat saturated cases and 12 non-fat saturated cases (70% fat saturated). The hold out test set had 14 fat saturated cases and 6 non-fat saturated cases (70% fat saturated).

### Preprocessing

The model was trained as a two-channel approach with the MR image slice as one channel, and the kidney segmentation as the other. Note that with this two-channel approach, the neural network learns to only identify cysts within the kidney. The images were rescaled to 256 × 256 matrix size using inter-cubic interpolation for the MR images, and nearest neighbor interpolation for the kidney and cyst segmentation masks. The intensity of each MR scan was first normalized to all have the same 95^th^ percentile level and then standard scalar normalization was applied (zero mean, unit standard deviation).

### Semantic segmentation model

The network architecture was similar to our previous works [22, 23]. The convolution blocks consist of 2D convolutions, followed by dropout (dropout = 0.1), batch normalization, 2D convolutions, and max pooling (pool size = 2 × 2). The higher -resolution layers have larger kernels (going from 7 × 7 to 5 × 5 to 3 × 3 in blocks down the encoder path, and in reverse up the decoder path) in order to learn larger and more complex filter types. The skip connections are implemented as additive layers (Resnet-like [24]). The optimizer is Adam [25] with an initial learning rate of 1e-3, and decay of 1e-5. The loss metric is the Dice similarity metric. The model is trained for 200 epochs with a batch size = 8 and the model with the best validation measure is saved during the training process. The model was implemented in Keras with TensorFlow as the backend. The model was trained on an Nvidia Tesla P40 GPU (24 GB memory). The input to the model is a two-channel matrix (256 × 256 × 2). The first channel is an MR image slice and the second is the corresponding kidney mask. The output is the prediction for the cyst segmentation. In total, three models were trained on the three different training/validation folds, and an ensemble, majority vote model was then made and applied to the hold out test set. Code is made available at: https://github.com/TLKline/AutoKidneyCyst.

### Evaluation

As described in the model section, the training/validation set was broken up into three folds in order to train on different subsets of the data. For each fold, training and validation curves were generated during the learning process and the best model from each fold was saved. A majority ensemble model was then generated and applied to the hold out test dataset. Comparison of cyst volume and cyst index was performed by linear regression, and cystic index was also assessed by Bland–Altman analysis in order to assess bias and precision of the measurements. In addition, visual overlays were made to qualitatively assess the automated method, and similarity metrics were generated for quantitative assessment. In each case, the two reader segmentations were compared in order to assess interobserver variability, and the automated approach was compared individually to each reader.

## Results

**Fig. 1:**
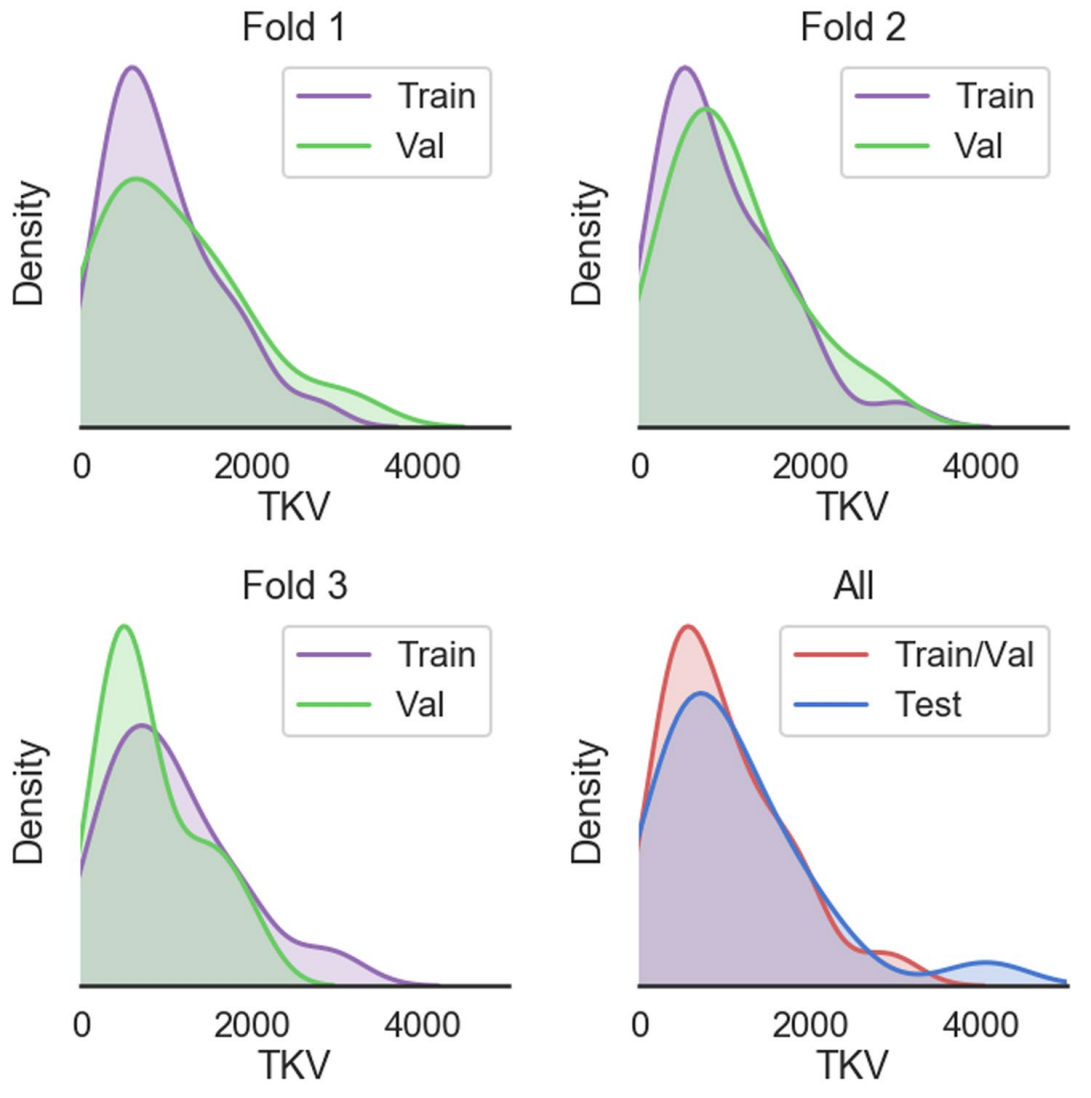
Visualization of density distributions of total kidney volume for the three folds (Fold 1: top left, Fold 2: top right, Fold 3: bottom left), and the entire training and validation sets as well as the separate hold out test set (bottom right). The cross-validation folds were randomly separated into the distinct subsets. The network model was trained on the three folds and an ensemble network was made and applied to the hold out test set

There was no significant difference between the training, validation, and testing datasets in terms of disease severity (i.e., TKV). Shown in Fig. 1 are the volume distributions visualized as kernel density plots. These are shown for the three folds, as well as the overall distribution between training/validation, and the test set. This overall distribution is representative of the large degree of variability seen in the ADPKD patient population.

**Fig. 2:**
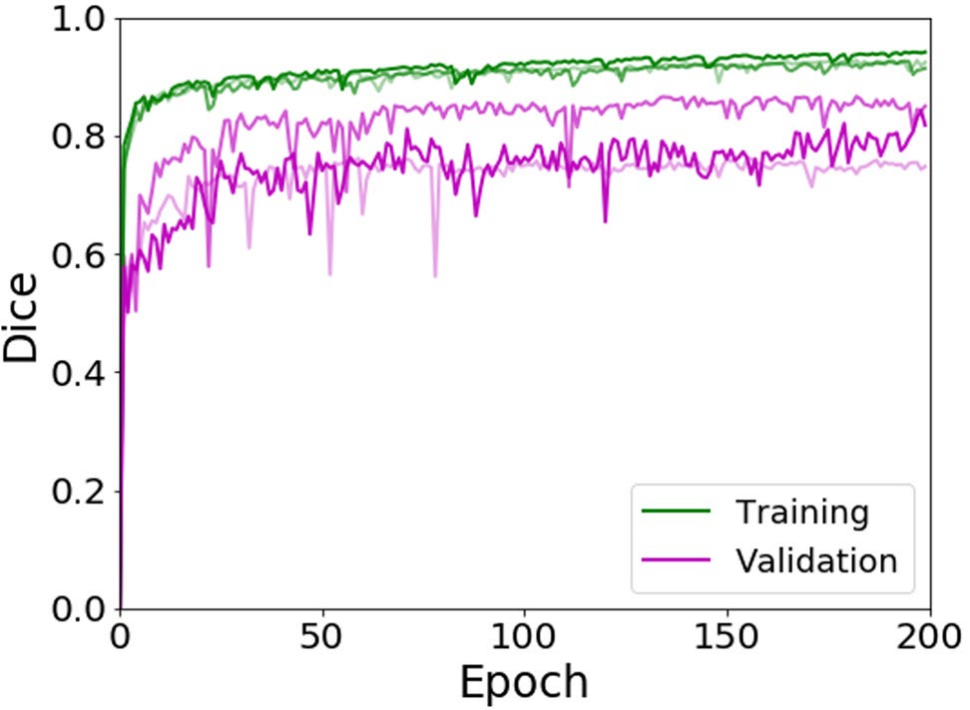
Learning curves for training and validation datasets from the three different folds. The darkness of the line corresponds to the higher fold number (i.e., Fold 3 is the darkest line). The Dice similarity metric was calculated at each epoch. In the case of fold 1, the final training Dice was 0.91 and validation was 0.76. In the case of fold 2, the final training Dice was 0.92 and validation was 0.87. For fold 3, the final training Dice was 0.94 and validation was 0.84

The automated method had similar performance training on the three different folds. Figure 2 shows the learning curves for the three different folds, including both training and validation Dice values during model training. The model weights are updated on the training set and evaluated at the end of each epoch on the separate validation set. The model with best validation performance is saved during the training process and used to develop the final ensemble model.

**Fig. 3:**
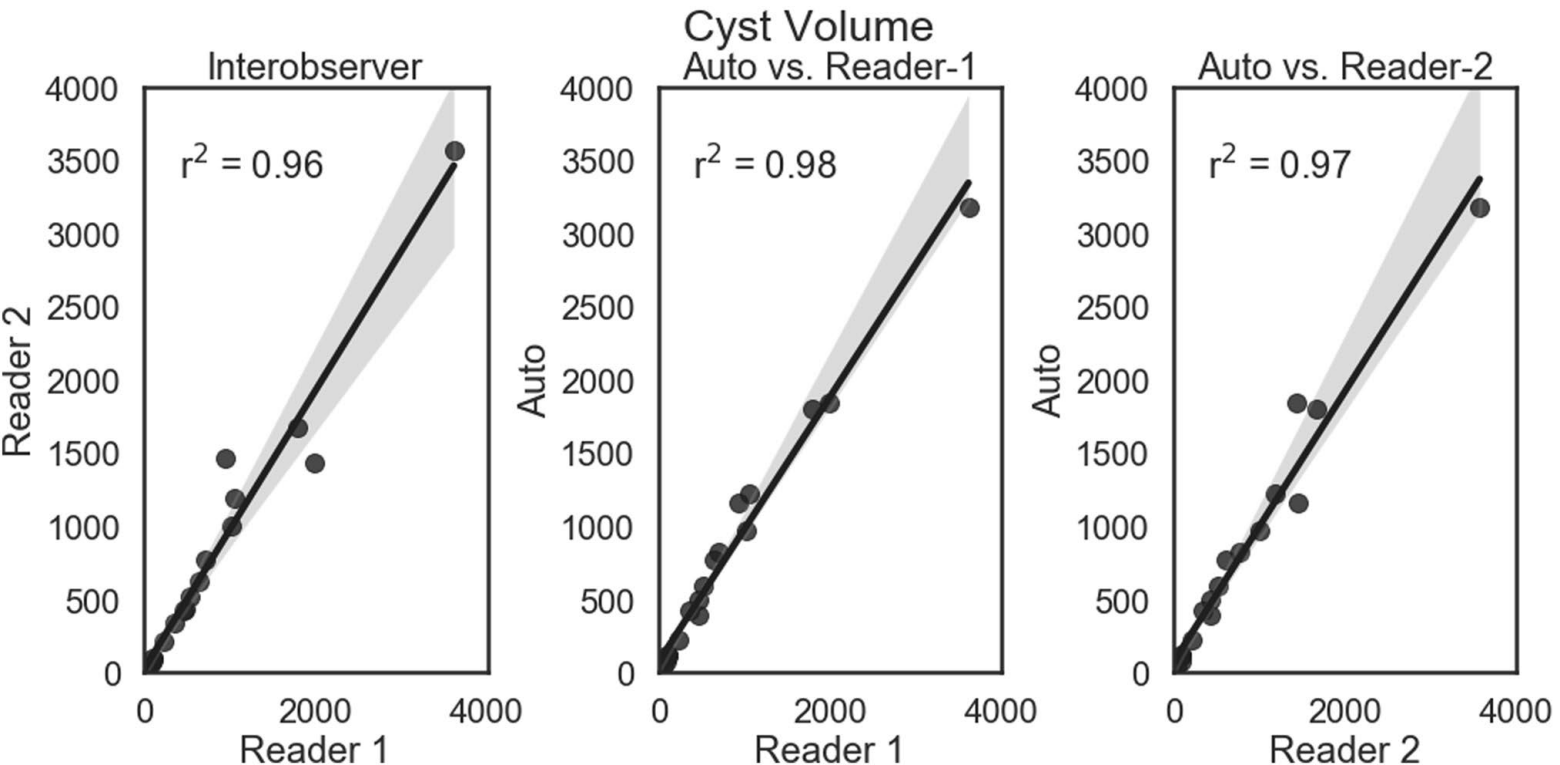
Linear regression comparisons for Cyst Volume. Comparisons are shown for interobserver (left panel), the automated method vs. Reader-1 (middle panel), and the automated method vs. Reader-2 (right panel). The automated approach performed very similar in the case of cyst volume with the two readers. The regression line is shown as a solid line (from the fit of *y* = *mx* + *b*) and the shaded region is the 95% confidence interval

**Fig. 4:**
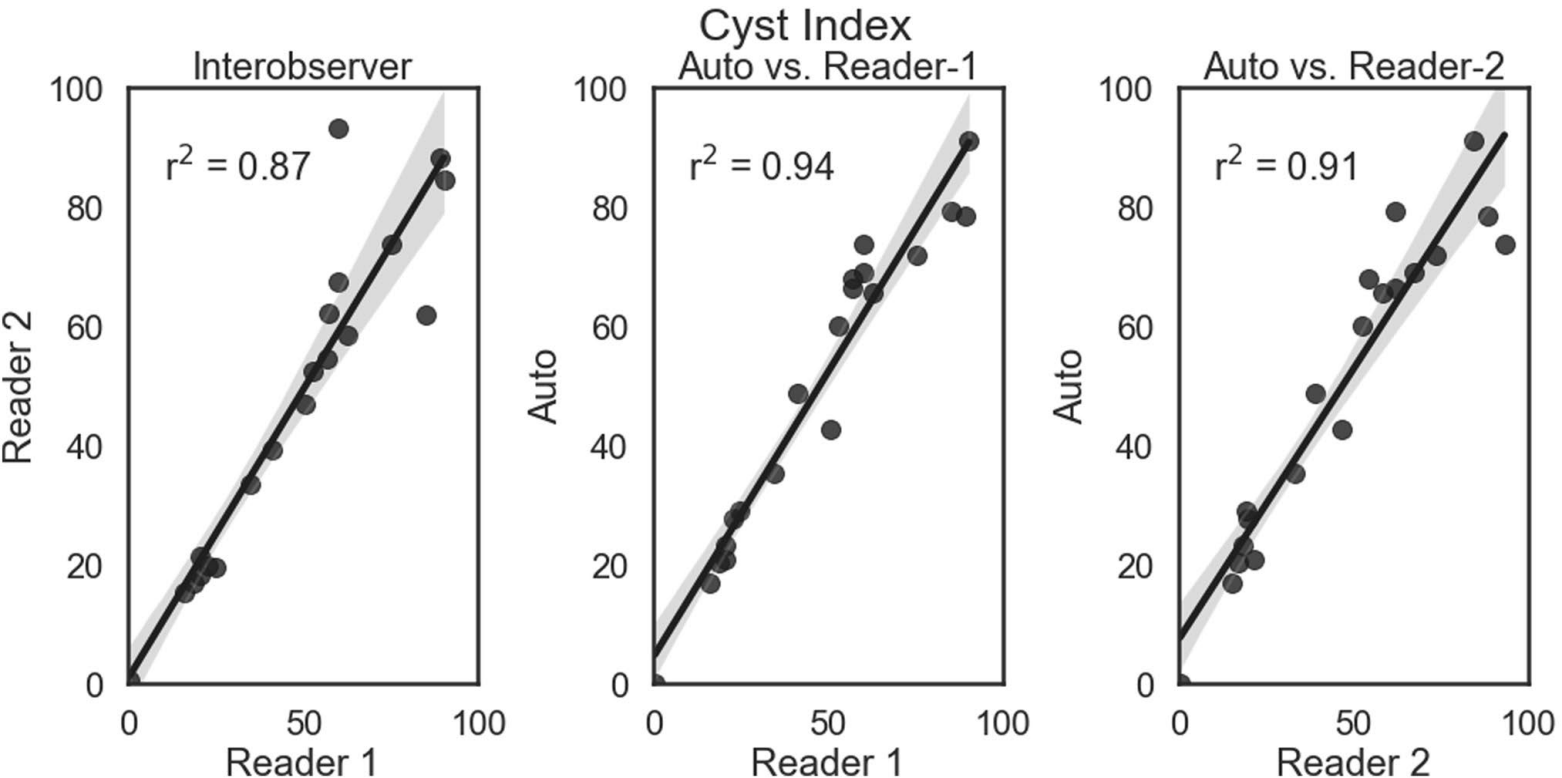
Linear regression comparisons for Cyst Index. Comparisons are shown for interobserver (left panel), the automated method vs. Reader-1 (middle panel), and the automated method vs. Reader-2 (right panel). The automated approach performed very similar in the case of cyst index compared with the two readers

**Fig. 5:**
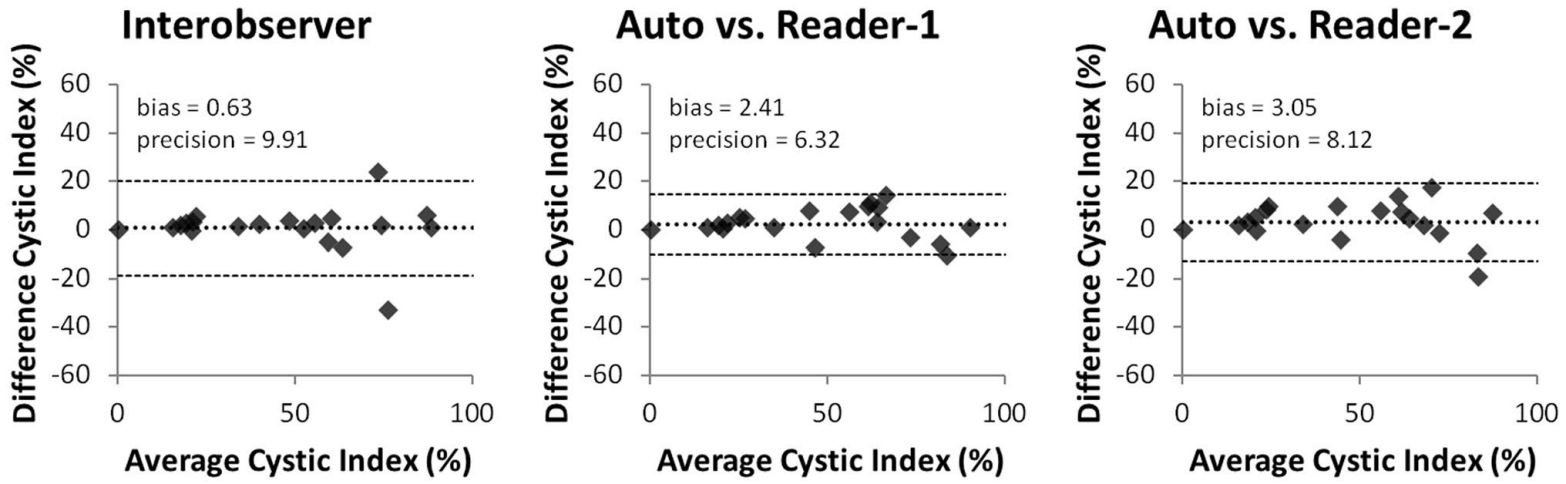
Bland–Altman results for the comparison of cystic index for interobserver (left panel), the automated method vs. Reader-1 (middle panel), and the automated method vs. Reader-2 (right panel). The two readers had very little bias between the overall measurements, but actually had a slightly larger precision than what was found for the automated method vs either reader independently

The automated approach was excellent at segmenting the cysts accurately. Shown in Figs. 3 and 4 are the linear regression comparisons for interobserver variability, the automated method vs. Reader-1, and the automated method vs. Reader-2 for cyst volume (Fig. 3), as well as cyst index (Fig. 4). In addition, the automated method performed at a similar level to that of human readers. Shown in Fig. 5 are the Bland–Altman comparisons for cystic index. Note that the patients encompass a wide range of disease severity, from cases with very few cysts, to cases will almost complete replacement of kidney parenchyma by cysts. The cystic index ranged from ~ 0 to > 90%.

**Fig. 6:**
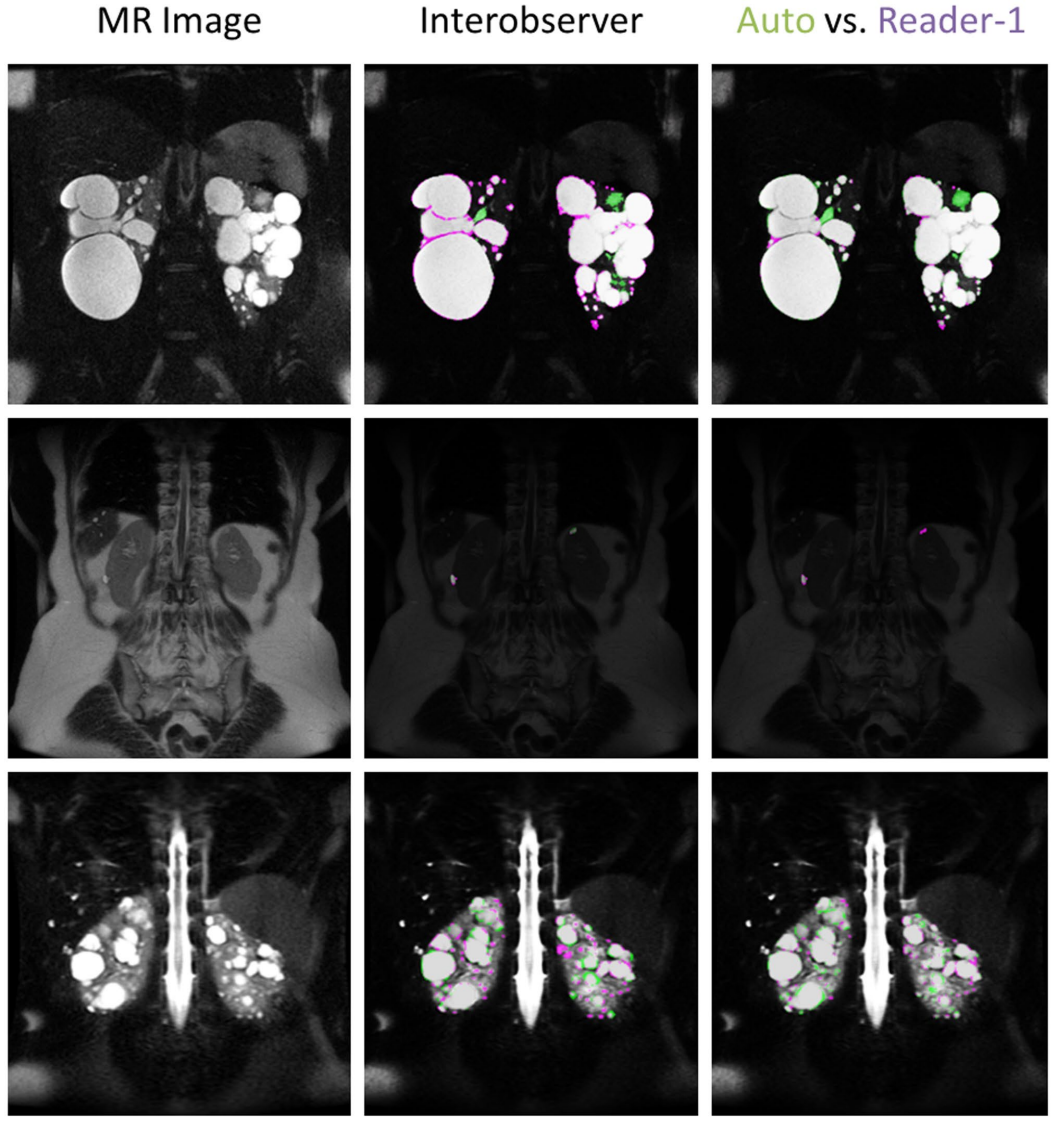
Visual comparisons between the interobserver segmentations and the automated approach compared to Reader-1. Shown in the left column are the MR images, the middle column are the gold-standard tracings comparing Reader-1 (violet) to Reader-2 (green), and right column compares Reader-1 (violet) to the automated approach (green). The top row highlights one of the best cases, with a Dice of 0.96 for interobserver, and 0.97 for the automated approach compared with Reader-1. The middle row is the worst case in terms of the automated methods performance, with an interobserver Dice metric of 0.66 and an automated Dice of 0.50 vs. Reader-1. The bottom row highlights a fairly typical case in terms of performance, with interobserver Dice of 0.84, and automated Dice of 0.86 vs. Reader-1. Regions that are seen to cause the greatest variability for both manual tracings as well as the automated approach are bright vessels, the renal pelvis, as well as complex cysts (appearing dark on the T2-weighted images). Agreement between the two is shown as dark gray/transparent. The background image is darkened in order to better visualize the segmentation overlap

Visually there was exceptional agreement between the automated segmentation approach and the manual readers. Figure 6 shows the visual comparisons for one of the better cases (top row, Dice = 0.98), the worst case (middle row, Dice = 0.50), and an average case (bottom row, Dice = 0.86).

In general, the automated approach was indistinguishable from the variability seen by two different readers performing the tracings. Shown in Table 1 are the similarity statistics comparing the interobserver variability to that obtained between the automated approach and Reader-1, as well as the automated approach and Reader-2.

**Table 1:** Segmentation metrics calculated for the two manual tracings, the automated approach vs. Reader-1, as well as the automated approach vs. Reader-2

Metric	Interobserver	Auto vs. reader-1	Auto vs. reader-2
Jaccard	0.76 ± 0.11	0.77 ± 0.13	0.74 ± 0.13
Dice	0.86 ± 0.08	0.86 ± 0.10	0.84 ± 0.11
Sensitivity	0.85 ± 0.08	0.88 ± 0.14	0.88 ± 0.15
Precision	0.88 ± 0.11	0.86 ± 0.07	0.83 ± 0.08
Hausdorff (vox)	7.58 ± 5.01	10.24 ± 4.86	17.87 ± 28.56
TCV Percent Difference	− 2.00 ± 16.40	3.87 ± 19.08	8.14 ± 24.06

Metrics were calculated from the cyst segmentation masks

## Discussion

Deep learning within the field of AI has provided scientists with countless tools for evaluating data efficiently and thoroughly, particularly in medical image analysis. The algorithm developed in this study accurately segmented renal cysts from kidney tissue without user intervention. Prior to this model, approaches to delineate cystic structures from organ tissue implemented semi-automated intensity-based thresholding techniques [16, 17, 20]. One limitation of intensity-based approaches is that, unlike CT, MR pixel values can drastically vary between acquisitions, and even between slices within one acquisition, requiring extensive preprocessing techniques to appropriately normalize the data [26]. Furthermore, this technique of intensity-based thresholding will completely miss complex cysts that have lower signal intensity [16].

The model presented in this study achieved a mean Dice score of 85% for cyst segmentation, this result is comparable to the other state of the art techniques implemented for organ segmentation. In ADPKD, all automated approaches using deep learning reported in the literature have focused on the organ segmentation task, mostly for kidney segmentation. Some of these approaches include, a customized VGG-16 network implemented by Sharma et. al [27] to segment kidneys in CT images. The average Dice score from this study was 86%. Keshwani et. al, [28] similarly used CT scans to predict kidney segmentations, a multi-task 3D convolutional neural network was implemented achieving a mean Dice score of 95%. Mu et al. [29], on the other hand, used MR images to automatically generate kidney segmentations using a V-Net model, and the reported Dice score was 95%.

The automated approach compared very closely to manual tracings in all metrics. In terms of linear regressions, the automated approach compared very closely to both of the readers. In addition, the cystic index had a similar bias and precision to human readers. The better precision is likely owed to the fact that the automated approach will be more consistent than a human reader. It was found that the largest difference was seen in the Hausdorff distance, which may be the result of some minor false positives which could likely be handled by simple post-processing (e.g., multiplying the output of the model’s cyst segmentation mask by the kidney mask). In addition, the visual agreement was incredibly strong. The worst case, in terms of similarity metrics, was for a very mild presentation of the disease. In this case, a human reader could quickly provide a quality assessment to finalize the cyst segmentation. In general the approach accurately segments cysts of a wide range of sizes. In this study, cysts were measured down to ~ 3-5 mm. This is limited by the reconstructed image resolution, which in-plane is on the order of ~ 1.5 mm. In addition, the largest cyst had a diameter of 118 mm.

Having the ability to automatically assess cystic burden opens up the door to retrospective studies applying the technique presented here. Prior studies have applied more basic approaches for assessing cystic burden and have shown the promising informative value of these image-derived parameters. Previous short-term studies have shown that tolvaptan decreased cyst volume in treated ADPKD patients when cyst volume was measured on a small cohort [30]. Further analysis should be completed to evaluate whether these effects continue throughout long-term administration of the drug. The automated method presented in this study will allow for quick and easy analysis of a larger dataset. Tracking cyst growth can also inform on specific genotypes. One study found that patients with PKD1 have a greater number of cysts than patients with PKD2. More specifically, patients with PKD1 progress faster because more cysts develop early on, not because they grow faster [11].

One limitation of this study is that it evaluated a relatively small cohort (*n* = 60). However, generating gold-standard cyst segmentations took up to 8 h depending on disease severity. Due to this limitation, we developed this particular cohort to span the full extent of disease phenotypic presentations, from kidneys composed of few cysts (cystic index = 0.5%) up to kidneys with renal parenchyma almost entirely replaced by cysts (cystic index = 90%). Having established a method to assess cystic burden over the full extent of disease phenotypes will make this approach strongly generalizable. Another limitation is that we are not detecting microscopic cysts below the imaging resolution. However, these microcysts contribute a relatively small amount to the total cyst volume [31]

Future studies can evaluate larger cohorts, and automated methods can be explored to segment and differentiate individual cysts. This will facilitate automatically counting the number of cysts and evaluating cyst size distributions. This may also allow for automatically classifying typical from atypical patients, which informs on risk of progression and likelihood to benefit from drug therapies. Most of the criteria that separate the atypical from the typical cases rely on cyst index, count, and size. For example, a patient is considered atypical if ≤ 5 cysts account for ≥ 50% TKV and there is mild replacement of kidney tissue from cysts [32]. A tool which calculates this automatically would allow for extremely fast and objective classifications during the critical study enrollment phase.

Cyst structure and composition are also seen as highly informative when assessing ADPKD. Once the cystic regions are delineated from the renal parenchyma, further intensity- and/or texture-based analysis may be performed to determine the percentage or distribution of complex cysts. Typically, these complex cysts are characterized by “darker” intensities in T2-weighted MR imaging. Seemingly, healthy parenchyma tissue can be analyzed in a similar way after being isolated from larger cysts. Another approach will be to incorporate multiple image acquisitions (e.g., combining T1- and T2-weighted MR images) in order to not only aid in the segmentation of cysts but also to help classify them as well. Extension to other imaging modalities (e.g., CT) and organs (e.g., liver) will also be important to provide a comprehensive characterization of the PKD phenotype and perform large-scale studies where mixed imaging data (e.g., ultrasound, computed tomography, and/or magnetic resonance imaging) are available for different patients, and extra-renal manifestations (e.g., PLD) are present.

## Conclusions

We have developed a fully automated method for semantic segmentation of kidney cysts from MR images of patients affected by ADPKD. The method performs on par with human readers and will be useful in future retrospective and prospective studies to evaluate patient phenotypes and overall cystic burden.

## Data Availability

Code is made available at: https://github.com/TLKline/AutoKidneyCyst
